# Therapeutic Versatility of Resveratrol Derivatives

**DOI:** 10.3390/nu9111188

**Published:** 2017-10-29

**Authors:** Waqas Nawaz, Zhongqin Zhou, Sa Deng, Xiaodong Ma, Xiaochi Ma, Chuangang Li, Xiaohong Shu

**Affiliations:** 1 College of Pharmacy, Dalian Medical University, Dalian 116044, China; imwaqasnawaz@yahoo.com (W.N.); dengsa31@163.com (S.D.); xiaodong.ma@139.com (X.M.); maxc1978@163.com (X.M.); 2 Academy of Integrative Medicine, Dalian Medical University, Dalian 116044, China; zzq0060@hotmail.com; 3 The Second Affiliated Hospital, Dalian Medical University, Dalian 116023, China

**Keywords:** resveratrol, resveratrol derivatives, pharmacological activity, structure-activity relationship

## Abstract

Resveratrol, a natural phytoalexin, exhibits a remarkable range of biological activities, such as anticancer, cardioprotective, neuroprotective and antioxidant properties. However, the therapeutic application of resveratrol was encumbered for its low bioavailability. Therefore, many researchers focused on designing and synthesizing the derivatives of resveratrol to enhance the bioavailability and the pharmacological activity of resveratrol. During the past decades, a large number of natural and synthetic resveratrol derivatives were extensively studied, and the methoxylated, hydroxylated and halogenated derivatives of resveratrol received particular more attention for their beneficial bioactivity. So, in this review, we will summarize the chemical structure and the therapeutic versatility of resveratrol derivatives, and thus provide the related structure activity relationship reference for their practical applications.

## 1. Introduction

**Figure 1:**
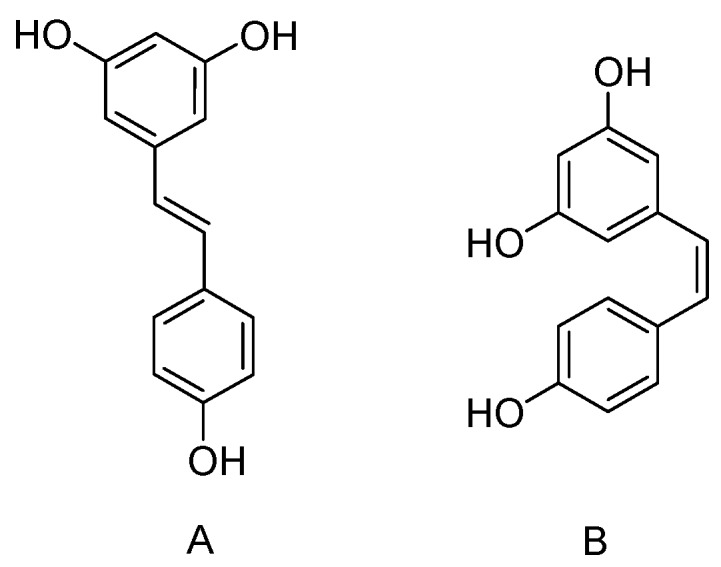
Chemical structures of *trans*-resveratrol (3,5,4′-trihydroxystilbene) and its *cis*-isomer. (**A**) *trans*-resveratrol; (**B**) *cis*-resveratrol.

Resveratrol (3,5,4′-trihydroxystilbene) is a natural phytoalexin produced by numerous plants in response to ultraviolet radiation, injury, fungal or bacterial infection. Resveratrol was first isolated from the roots of white hellebore (*Veratrum grandiflorum O*. Loes) in 1940 [1,2], and is well known for its “French Paradox” [2]. However, resveratrol not only exists in red wine but is present in natural foods including grape, blueberry, raspberry and mulberry, and the exact concentration of resveratrol in each source varies widely [3]. In the last few decades, stilbene-based compounds are widely investigated due to their diverse range of biological activities [4]. The chemical structure of resveratrol is composed of two aromatic rings allied by a methylene bridge, and presents in both *trans-* and *cis*-isomeric forms naturally (Figure 1), however, the majority of the recorded medical advantages are attributed to *trans*-resveratrol but not its *cis*-form, so the structure activity relationship is the key point to determine the biological activities of resveratrol [5,6].

A large number of preclinical studies have proved that resveratrol could impact a variety of human pathophysiological conditions [7]. The latest search (on 19 June 2017) from the database of the new ClinicalTrials.gov (http://clinicaltrials.gov/) shows that there are 104 clinical trials (statuses including active, recruiting, enrolling by invitation and completed) involving in resveratrol, and these trials aim to investigate the pharmacological activity of resveratrol in treating cancer, diabetes, obesity, and other cardiovascular and neurodegenerative diseases. The safety and tolerability of resveratrol as a medicinal product are investigated in the clinical trials [8,9]. It is extremely encouraged that doses of resveratrol of up to 5 g/day, taken for a month, are safe and well tolerated [9,10]. In humans, resveratrol could be absorbed after oral administration, but the plasma concentration and tissue distribution are low for its rapid and extensive phase II metabolism [10,11,12,13]. So, the search for a method how to increase the bioavailability of resveratrol receives more attention.

Resveratrol is a small molecule with a molecular weight of 228.247 g/mol, and it is equipped with different functional groups including phenolic hydroxyl groups, aromatic rings and a double bond [14]. These functional groups provide resveratrol with great potential to be modified into active derivatives with more diversified therapeutic efficacies [15,16,17]. In the past few years, researchers have synthesized a series of resveratrol derivatives and evaluated their biological activities. Pan et al. found that *trans*-3,4′,5-trimethoxystilbene showed higher potency than resveratrol in inhibiting angiogenesis, and it also possessed the potent proapoptotic activity [18]. Locatelli et al. synthesized 4,4′-dihydroxy-*trans*-stilbene, which showed strong antioxidant properties [19]. Das et al. synthesized various monoalkoxy and dialkoxy derivatives of resveratrol to improve Protein kinase C (PKC) binding activity, which was proved to be more effective than the parent compound [20]. Many studies showed pterostilbene possess better chemotherapeutic properties than resveratrol via in vitro and in vivo experiments [21].

The structure-activity relationship (SAR) studies have revealed that the parent structure of resveratrol is an essential component and is vital for its specific therapeutic effects [15]. Substitution of hydroxyl groups of resveratrol to methoxy groups significantly boosted its cytotoxic activity [22], and the hydroxy group in the *trans* conformation at the 4- and 4′-positions of the stilbene structure has been reported as essential for the antiproliferative effect of resveratrol [23]. The introduction of halogen groups could provide the antibacterial activity of resveratrol [24]. In addition, there are various naturally occurring and synthetic stilbene-like compounds which show different bioactivity but are related to their chemical structure. So the designing and synthesizing of novel resveratrol derivatives have received much more of attention from pharmacologists and thus to enhance the therapeutic versatility of resveratrol. During the last few decades, various natural and synthetic resveratrol derivatives were studied, and the methoxylated, hydroxylated and halogenated derivatives of resveratrol were received the special attention due to their diverse therapeutic potential. This review aims to highlight the role of the resveratrol derivatives such as methoxylated, hydroxylated and halogenated derivatives, and focus on their therapeutic versatility and their action mechanism.

## 2. Methoxylated Derivatives of Resveratrol

SAR shows that the substitution of hydroxyl groups of resveratrol to methoxy groups substantially potentiated its therapeutic versatility [22]. Therefore, the researchers take a keen interest in synthetic and natural methoxylated analogs of resveratrol to explore their role in the treatment of various diseases.

### 2.1. Pterostilbene (3,5-Dimethoxy-4′ Hydroxystilbene) 

**Figure 2:**
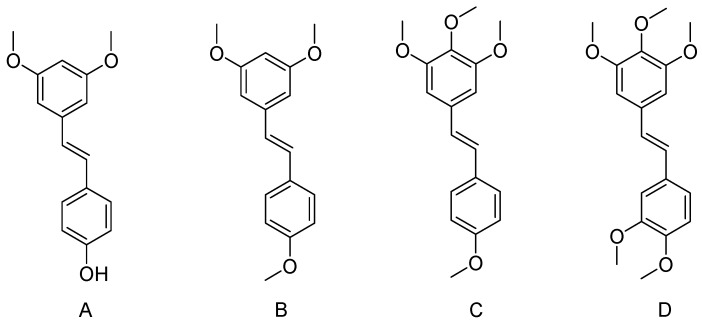
Chemical structures of methoxylated derivatives of resveratrol. (**A**) Pterostilbene; (**B**) Trimethoxystilbene; (**C**) Tetramethoxystilbene; (**D**) Pentamethoxystilbene.

Pterostilbene ((*E*)-3,5-dimethoxy-4′-hydroxystilbene, Figure 2A) is chemically classified as a benzylidene compound. The substitution of hydroxy with methoxy groups enhances the lipophilicity of pterostilbene, adding to its in vivo bioavailability, and thus improves the biological activity of pterostilbene compared to the parental compound resveratrol [25]. Pterostilbene has been reported to exert various pharmacological effects including antioxidant, anticancer, cardioprotective, neuroprotective and antidiabetic properties [21]. Substituting the hydroxyl for the methoxy group could enhance the structure stability of the compound, and pterostilbene is not as quickly glucuronidated and sulfated as resveratrol, and thus possess higher bioactivity than resveratrol. A study evaluated the preclinical pharmacokinetics and pharmacodynamics was performed on the male Sprague Dawley rats dosed i.v. with 20 mg/kg of *trans*-pterostilbene, and then the samples were analyzed by high performance liquid chromatograpy (HPLC). The results showed that the pterostilbene glucuronidated metabolite could be detected in both serum and urine, but the area under the plasma concentration time curve (AUC), serum t(1/2), urine t(1/2) of pterostilbene were 17.5 ± 6.6 μg/h/mL, 1.73 ± 0.78 h, 17.3 ± 5.6 h, respectively [26]. Another pharmacokinetic study in rats showed the terminal elimination half-life and clearance of pterostilbene were 96 ± 23 min and 37 ± 2.5 mL/min/kg, respectively. And its absolute oral bioavailability was 12.5 ± 4.7% [27]. From above studies, pterostilbene showed improved pharmacokinetic characteristics compared to its naturally occurring analog, resveratrol.

#### 2.1.1. Antioxidant Activity

Rimando et al. evaluated the antioxidant potential of pterostilbene for the first time, and they found thatpterostilbene showed similar free radical scavenging activities to that of resveratrol [28]. Remsberg et al. studied the antioxidant effect of resveratrol, pterostilbene, quercetin and their combinations in human erythrocytes, which showed that pterostilbene protecting erythrocyte membranes against hydrogen peroxide-induced lipid peroxidation [26]. Hasiah et al. studied the total antioxidant activity of methoxylated resveratrol derivative on HepG2 hepatoma and Chang liver cells by adopting ferric reducing antioxidant power assay, and found that pterostilbene showed strong antioxidant activity [29]. The researchers also found that pterostilbene could regulate several signals including nuclear factor kappa-light-chain-enhancer of activated B cells (NF-κB), Forkhead box class O family member proteins (FoxOs), nuclear factor erythroid 2-related factor 2 (Nrf2)/Kelch-like ECH-associated protein 1 (Keap1) and activator-protein-1 (AP-1), and thus indirectly exerted the antioxidant activities [30].

#### 2.1.2. Chemotherapeutic Activity

Some studies reported that pterostilbene could play an important role in chemotherapy through various mechanisms, including inflammation, angiogenesis, radiation resistance and acting on multiple targets [31,32,33,34,35,36,37,38,39]. Pterostilbene decreased cancer cell viability in different human cancers including breast cancer, pancreatic cancer, lung cancer, gastric cancer, melanoma and leukemia [31]. Studies showed that pterostilbene inhibited aberrant crypt foci formation and cell proliferation in colon cancer models. It was reported that pterostilbene could reduce the gene expression of Myc, beta-catenin and cyclin D in human colon adenoma cells [32].

Pterostilbene inhibited tumor in human hepatocellular carcinoma cells by suppressing various signal transduction pathways [33], including mitogen-activated protein kinase (MAPK), NF-κB, matrix metalloproteinase-9 (MMP-9), expression of vascular endothelial growth factor (VEGF) and AP-1 [34]. Similarly, it also suppressed the gastric adenocarcinoma cells by inhibiting cellular proliferation and apoptosis via different mechanism, including alteration of cell-cycle regulating proteins and activation of the caspase cascade [35].

Pterostilbene was reported to induce cell apoptosis and inhibit cell viability in breast cancer cell lines. Wu and coworkers found that pterostilbene selectively inhibited CD44^+^/CD24^−^ cancer stem cells in MCF-7 cells [34,36]. Pterostilbene treatment increase the chemotherapy sensitivity to breast cancer stem cells (BCSCs), and reduced CD44 expression of BCSCs and enhance phosphorylation of β-catenin [34]. Pterostilbene inhibited the cell growth of melanoma cells through inhibition of vascular cellular adhesion molecule-1 (VCAM-1) expression. It was proven to be effective against melanoma in combination with quercetin synergistically. It suppressed the carcinogenesis in mouse epidermis by inhibiting the 12-*O*-tetradecanoylphorbol-13-acetate (TPA), NF-κB and AP-1 activation [37]. Recent studies showed that pterostilbene could down-regulate the phosphatase and tensin homolog (PTEN)-targeting members of the oncogenic miR-17 family, which were over-expressed in prostate cancer, and functionally validated the miRNA-mediated ability of resveratrol and pterostilbene to rescue the tumor suppressor activity of PTEN as therapeutic strategy [38].

#### 2.1.3. Cardioprotective Activity 

The cardioprotective role of resveratrol and its derivatives received more attention for the “French Paradox”. It was reported that nitric oxide (NO), prostacyclin, Angiotensin II, endothelin-1 and thromboxane A2 played a vital role in regulating the diameter of the vessel and thus regulating blood pressure. Pterostilbene could enhance endothelial NO production, and result in endothelium-dependent relaxation in hypertension and stroke [40]. Therefore, it has been proposed that the consumption of a certain level of pterostilbene would be beneficial to keep the health of cardiovascular system. Molecular docking analysis showed that pterostilbene could bind to the enzymatic active pocket of sirt1 and stimulate sirt1 activity, enhance the expression of sirt1. The present study demonstrated that pterostilbene exerted its cardioprotective effects via activation of sitr1 during ischemia reperfusion [41]. Importantly, pterostilbene rescued H9c2 cells from apoptosis at low concentrations of 0.1–3.0 μM, an effect comparable with 20 μM resveratrol. So, pterostilbene could be used to alleviate ischemia-reperfusion injury therapeutically.

Human clinical trials showed that resveratrol could improve left ventricle diastolic function, endothelial function, lowering low density lipoprotein (LDL)-cholesterol level and protecting against unfavorable hemorheological changes measured in patients with coronary artery disease (CAD) [42]. Pterostilbene stimulated cytoprotective macroautophagy in vascular endothelial cells and protected vascular endothelial cells against oxidized low-density lipoprotein-induced apoptosis [40]. Furthermore, a clinical trial at University of Mississippi Medical Center USA demonstrated that a pTeroPure (a patented Irvine, patent number: 8,133,917, Chromadex, 10005 Muirlands Blvd, Irvine, CA 92618, USA) compound that corresponds to a pterostilbene plays a key role in hypertension management in humans (www.clinicaltrials.gov). These findings recommended further research on pterostilbene to highlight and specify its role in cardiovascular diseases.

#### 2.1.4. Neuroprotective Activity

Recent studies have shown pterostilbene exerted a favorable effect in the protection against age-related diseases including Alzheimer’s disease (AD). Pterostilbene has been reported to protect cells from oxidative stress, aging and dysregulation of autophagy via acting on various signaling pathways and protein homeostasis, and which can significantly contribute to restore cognitive function during the aging process [43]. Chang et al. demonstrated that two months of pterostilbene diet significantly improved radial arm water maze function in SAMP8 (Alzheimer’s/aging mouse model) compared with control-fed animals. Importantly, the markers of inflammation, cellular stress and AD pathology were positively modulated by pterostilbene but not resveratrol, and which were associated with upregulation of peroxisome proliferator-activated receptor (PPAR) alpha expression [44]. MnSOD, an important oxygen free radical scavenger, could resist oxidative stress, and thus exerts key roles in neuroprotective function. It was also reported that pterostilbene could enhance the manganese superoxide-dismutase (MnSOD) expression in SAMP8 [44], and pterostilbene as an active antioxidant responsive element inducer might be the potential reason for upregulating the expression of MnSOD [45]. So, the findings indicates that pterostilbene is a more potent modulator of cellular stress and cognition than resveratrol.

Treatment with pterostilbene has also been demonstrated to protect neurons from neuro-inflammation via the inhibition of reactive oxygen species (ROS) production and the inactivating of NF-κB signaling pathways. Furthermore, it inhibits activation of SIRT1 pathways and MAPK signal transduction pathways [46]. Numerous studies show that pterostilbene has significant effects on cognition and neuronal function during aging. Joseph et al. demonstrated that pterostilbene administration in aged rats improved hippocampus functions characterized by behavior and biochemical assays [47]. Furthermore, it maintains the oxotremorine augmentation of dopamine production from striatal slices. In another study of accelerated SAMP8 mice model, pterostilbene was found to be more effective and preserve cognitive function than resveratrol [44]. Pterostilbene shows better activity than parent compound due to its potential of crossing the blood brain barrier and intracellular calcium buffering capacity [44].

#### 2.1.5. Antidiabetics 

Amarnath and Pari studied the pterostilbene antioxidant role in type 2 diabetes mellitus rat model, and after a dose of 40 mg/kg pterostilbene treatment for six weeks, the levels of GSH peroxidase, superoxide dismutase, GSH S-transferase were significantly improved in liver and kidney of diabetic animals, but the increased levels of lipid peroxidation in liver and kidney of diabetic rats, which measured as thiobarbituric acid reactive substances (TBARS), became normalized. In addition, the pathological changes observed in the liver and kidney of diabetic rats were significantly reduced after chronic pterostilbene treatment [48]. It was reported that hypercholesterolemic hamsters fed with pterostilbene at 25 ppm of the diet, and found 29% lower plasma LDL cholesterol, 7% higher plasma high density lipoprotein (HDL) cholesterol, and 14% lower plasma glucose as compared to the control group. The in vitro studies showed that pterostilbene might be a more effective PPAR-α agonist and hypolipidemic agent than resveratrol. And the in vivo studies also demonstrated that pterostilbene exerted lipid and glucose lowering effects [49]. These results indicated that pterostilbene would be a favorite candidate antidiabetic drug.

### 2.2. Trimethoxystilbene 

*Trans*-3,4′,5-trimethoxystilbene (3,4′,5-TMS) (Figure 2B) is a methylated resveratrol derivative and is reported to be most potent proapoptotic, antioxidant and vascular-targeting analog of resveratrol. It was first reported by Blair G.E. and coworkers in 1969 [50]. 3,4′,5-TMS exist in E and Z isomeric form. Pharmacokinetic studies show that they also had similar long terminal elimination half-lives (t1/2: 395 ± 121 min vs. 366 ± 104 min). E isomer has more tissue distribution and therefore it has less plasma exposure and larger volume of distribution. However, Z isomer may have less tissue distribution and therefore it has more plasma exposure and smaller volume of distribution. Clearly, the distribution profiles of 3,4′,5-TMS were dependent of the E- and Z-isomers. The oral bioavailability of Z isomer was problematic. The oral pharmacokinetics of E-TMS was more favorable than the Z-isomer. After oral gavage, E isomer was absorbed rapidly with an absolute bioavailability of 54.9 ± 28.1% [50]. In comparison with resveratrol, 3,4′,5-TMS had greater plasma exposure, longer elimination half-life and lower clearance. As 3,4′,5-TMS had superior pharmacokinetic characteristics, its potential as a preventive or therapeutic agent in resveratrol-effective conditions or diseases should be considered [51].

#### 2.2.1. Chemotherapeutic Activity 

*Trans*-3,4′,5-trimethoxystilbene (3,4′,5-TMS) has an active anticancer profile than resveratrol, as proved from multiple pieces of literature describing a strong induction of cell cycle arrest, reduced angiogenesis, cancer cell proliferation inhibition, increased apoptosis and decreased metastasis. It suppressed the breast cancer by acting on the MCF-7 cells which inhibited the activity of epithelial–mesenchymal transition (EMT). 3,4′,5-TMS showed its action by down-regulating phosphatidylinositol 3-kinase (PI3K)/protein kinase B (AKT) signaling, and β-catenin nuclear translocation and thus inhibits invasion of breast cancer cells [52]. In lung cancer cell lines, 3,4′,5-TMS induced apoptosis by caspase-9 and caspase-3 activation and blocking poly (ADP-ribose) polymerase, reducing the potential of the mitochondrial membrane and increased B-cell lymphoma 2-associated X protein (Bax)/B-cell lymphoma 2 (Bcl-2) ratio [53]. Yang et al. studied the anti-metastatic potential of 3,4′,5-TMS on human lung cancer A549 cell line [54]. Their results showed 3,4′,5-TMS inhibited the migratory, invasive and adhesive properties of the A549 cancer cell line and mRNA levels of the MMP-2 protein. G. Wang et al. demonstrated 3,4′,5-TMS to be more effective than resveratrol in suppressing the growth of HepG2 hepatocellular carcinoma cells by induction of G2/M cell cycle arrest (upregulation of Cyclin B1) and apoptosis (downregulation of Bcl-2) [55].

#### 2.2.2. Cardioprotective Activity 

3,4′,5-TMS significantly prevented the pulmonary vascular remodeling and right ventricular hypertrophy. It could decrease the wall thickness of vessels and right ventricle weight, which was characterized by downregulation of VPO1 and NOX2, NOX4 expression in right ventricle and/or pulmonary artery and reduce H_2_O_2_ synthesis [56]. 3,4′,5-TMS had a significant role in treating pulmonary arterial hypertension, it had strong inhibitory effects on the growth of pulmonary artery smooth muscle cells as compared to resveratrol. It could attenuate the progress of pulmonary arterial hypertension and induce apoptosis in pulmonary artery smooth muscle cells [57]. 3,4′,5-TMS also regulated the macroautophagy, which could control the function of vascular endothelial cells. It could induce autophagy by increasing the expression of the transient receptor potential canonical channel 4 and plasmid transfection. Intracellular Ca^2+^ concentration increased due to upregulation of TRPC4, which could activate the Ca^2+^/CaMKK β/AMPK pathway and led to autophagy [58]. 3,4′,5-TMS caused intersegmental vessel regression, normal stasis of blood flow and inhibited blood vessel growth in a zebrafish model. It downregulated the expression of vascular endothelial growth factor 2 (VEGFR2) and caused G2/Mcell-cycle arrest [59]. Belleri et al. reported that the vascular-targeting potential of 3,4′,5-TMS was 30 to 100 times more potent than the resveratrol in inhibiting endothelial cell proliferation, sprouting, collagen gel invasion, and morphogenesis [60].

#### 2.2.3. Anti-HCV Activity

Nguyen et al. studied the anti-HCV (Hepatitis C virus) effect of 3,4′,5-TMS in an HCV infection model of Huh7.5 cells [61]. The infected cells were treated with 3,4′,5-TMS the JFH1 RNA copy number was drastically ten times reduced with 3,4′,5-TMS treatment as compared with the dimethysulfoxide treated and untreated cells. 3,4′,5-TMS induced bundling of microtubules and speckle-like structures of double-cortin-like kinas1 (DCLK1) microtubule complexes, whose regulations were critical for HCV replication, cell division, cell migration, and cell polarity. Furthermore, it also inhibited the growth of GS5 cells as visible cell spheroids/aggregates and targeted the HCV positive DCLK1 overexpression. 3,4′,5-TMS had a great potential to be an anti-HCV drug, as it exerted its effects by multiple pathways and in a unique mechanism.

### 2.3. Tetramethoxystilbene 

*Trans*-3,4,5,4′-tetramethoxystilbene (3,4,5,4′-TMS) exhibited superior availability compared to resveratrol. Studies showed that resveratrol was metabolized to its sulphate or glucuronate conjugates, while 3,4,5,4′-TMS underwent metabolic hydroxylation or single and double *O*-demethylation. In the light of the superior levels achieved in the gastrointestinal tract after the administration of 3,4,5,4′-TMS when compared to resveratrol, the results provide a good rationale to evaluate 3,4,5,4′-TMS as a good therapeutic target [62]. Another study showed that 3,4,5,4′-TMS intravenous administration in rats show moderate clearance (46.5 ± 7.6 mL/min/kg) and terminal elimination half-life (154 ± 80 min). However, the absolute oral bioavailability of 3,4,5,4′-TMS was low (6.31 ± 3.30%) [63]. Pharmacokinetic studies in mice revealed that higher levels of 3,4,5,4′-TMS compared to resveratrol were achieved [64].

#### 2.3.1. Chemotherapeutic Activity

*Trans*-3,4,5,4′-tetramethoxystilbene (3,4,5,4′-TMS, Figure 2C), a resveratrol derivative, showed more strong bioactivity than the parent resveratrol in suppressing the growth of various cancer cell lines, including colon, prostate, ovarian cancer cells and hepatocellular. 3,4,5,4′-TMS (Figure 2) was a significant therapeutic tool for the treatment of human melanoma cells, while in A375 human melanoma, 3,4,5,4′-TMS suppressed the proliferation of A375 cells (IC_50_ = 0.7 µM) through mitotic arrest at the prometaphase stage of cell division [65]. It increases the expression of the mitogen activated protein kinases, p38 and Jun *N*-terminal kinase (JNK), activation of p38 and inhibited the localization of the protein to the spindle poles [65,66]. 3,4,5,4′-TMS inhibited the angiogenesis, by directly inhibiting VEGFR2-mediated signaling cascades and VEGFR2 activation in endothelial cells. It shows its action by inhibiting the phosphorylation of multiple downstream signaling components of VEGFR2, including Akt, FAK, c-Src, mTOR, p70S6K and extracellular signal-regulated kinase 1/2 (Erk1/2). It showed that 3,4,5,4′-TMS exerted its anti-angiogenic activity by suppressing VEGFR2 activation [67].

Regarding the chemotherapeutic property of tetramethoxystilbene, SAR showed that the *cis* form of 3,4,5,40-tetramethoxystilbene (i.e., 3,4,5,40-tetramethoxy-*cis*-stilbene) is ten times more potent than the *trans* isoform in suppressing the growth of human WI38VA virally-transformed fibroblasts. 3,4,5,4′-TMS was reported to evoke apoptosis in both mitochondria and the receptor-mediated manner in A-2780 and SKOV-3 (ovarian cancer cell lines). Treatment with tetramethoxystilbene for one week shows a significant decreases in the tumor growth of ovarian cancer [68]. Similarly, it was reported to suppress the growth of breast cancer cell by inducing mitochondrial apoptosis through the regulation of proapoptotic proteins and VDAC1 expression [69].

3,4,5,4′-TMS was a good candidate as a chemotherapeutic agent against hepatocarcinogenesis. 3,4,5,4′-TMS administration in N-nitrosodiethylamine animals reduced the level of inducible nitric oxide synthase (iNOS) and Signal transducer and activator of transcription 3 (STAT3) activation, and enhanced the constitutive AP-1 subunits c-Jun, c-Fos levels and c-Jun binding to TRE consensus site [70]. Analysis of *cis*-isomers of various *trans*-stilbene derivatives to explore SAR revealed that the *cis* isoform of 3,4,5,4′-tetramethoxystilbene (i.e., 3,4,5,4′-tetramethoxy-*cis*-stilbene) was ten times more potent than the *trans* isoform in inhibiting the growth of human WI38VA virally transformed fibroblasts [71].

#### 2.3.2. Effect on Activity of Cytochromes P450

Targeting cytochromes P450 was the best strategy for the therapeutic application in various disorders, including cancer, hypertension, various metabolic diseases, etc. Chun et al. [72] first reported 3,4,5,4′-TMS as a novel selective and strong inhibitor of human Cytochrome P450 1 (CYP1B1). 3,5,2′,4′-tetramethoxy-*trans*-stilbene possessed powerful potent inhibitory activity (IC_50_ = 2 nM ) toward CYP1B1 among other derivatives of polyphenol [73].

Various studies highlighted the interactions between tetramethoxystilbene and the AhR-CYP1A signaling pathway and define as substrates and inhibitors of CYP1A1, CYP1A2 and CYP1B1 enzymes [73,74]. A series of stilbene derivatives were synthesized to study their role in inhibiting human recombinant cytochrome P450 (CYPs): CYP1B1, CYP1A1, CYP1A2. Among all the studies, compound tetramethoxystilbene was found to be more potent and selective in inhibiting CYP1A1, CYP1A2 and CYP1B1 activities [75].

#### 2.3.3. Cardioprotective Activity

Angiotensin II (Ang II) and arachidonic acid (AA) could induce hypertrophy and vascular smooth muscle cell migration. 3,4,5,4′-TMS was found to inhibit AA and Ang II induced ROS generation and ERK1/2 [75]. 3,4,5,4′-TMS administration was found to reduce cardiac hypertrophy and cardiac fibrosis, which was characterized by the decrease in the brain natriuretic peptide (BNP), α-smooth muscle actin-positive cells and macrophages in the myocardium [76,77]. It could manage hypertension by inhibiting the activation of cPLA2 and release AA, which could generate ROS through CYP1B1. Previous studies also showed that hypertension induced due to other vasoconstrictor agents, phenylephrine, ET-1 and Ang II could be inhibited after treatment with 3,4,5,4′-TMS. Furthermore, it also treated hypertension in renal dysfunction induced by DOCA-salt and Ang II infusion [78].

3,4,5,4′-TMS led to inhibition of signaling molecules, including ERK1/2, p38 MAPK, and c-Src. The activation of these molecules led to ROS produced through CYP1B1, which could cause various cardiovascular diseases, including cardiovascular hypertrophy, endothelial dysfunction, proteinuria, enhance vascular reactivity, renal dysfunction, injury and inflammation [79].

### 2.4. Pentamethoxystilbene 

Pentamethoxystilbene (PMS), a hybrid molecule, was synthesized chemically. The pharmacokinetics of PMS was subsequently studied in rats. Upon intravenous administration (0.75 mg/kg), PMS showed moderate clearance (58.5 ± 19.5 mL/min/kg) and terminal elimination half-life (147 ± 61 min). Aqueous solubility appeared to be a barrier to oral absorption. When the suspension was given (4 mg/kg), the absolute oral bioavailability was almost nil; when PMS was fully solubilized by randomly methylated–cyclodextrin, it possessed a low bioavailability (3.63 ± 2.06%). The pharmacokinetic comparison among PMS and other methoxylated stilbenes suggested that the 2-methoxy group was unfavorable to oral bioavailability [80].

#### 2.4.1. Chemotherapeutic Activity

Pentamethoxystilbene (PMS, Figure 2D), a synthetically methoxylated analog of resveratrol, has high intravenous bioavailability, however, its oral bioavailability is low. PMS exhibited a strong antiproliferative effect by acting on multiple cellular signaling pathways which targeted breast carcinoma cells (MCF-7). It controlled several G1 cell cycle regulators, such as E- and D-type cyclins, Cyclin-Dependent Kinase Inhibitors (CKIs), Cyclin-Dependent Kinases (CDKs), and Phosphorylation of the retinoblastoma protein (pRb). PMS proved to be more potent in suppressing the growth of breast cancer cells than other methoxylated derivatives and resveratrol [81]. PMS was a strong inducer of apoptosis in colon cancer cells via targeting microtubules [82], and which showed a more potent antimitogenic effect on colon cancer cells than resveratrol. PMS initiated G(2)/M mitotic arrest and polymerization of microtubules, and led to caspase-dependent apoptosis. Furthermore, its suppressed colitis-related colon carcinogenesis by promoting apoptosis, inhibiting iNOS, cell proliferation and β-catenin inactivation [83].

The exact mode of action of PMS was unknown. However, it was supposed that it caused G1 cell-cycle arrest and G1 cell-cycle regulatory proteins. Which inhibited the expression of cyclin D3, D1 and E and cyclin-dependent kinases (CDK2, 4 and 6). The expression of CDK inhibitors, including p15, p16, p21 and p27 was enhanced. Furthermore, PMS promoted cell-cycle progression by activating several kinases, including Akt, MAPK, ERK1/2and focal adhesion kinase. It also regulated apoptosis by enhancing the phosphorylation of p38 MAPK [81].

#### 2.4.2. Cytochrome P-450 Inhibitor

PMS was reported to be a potent and selective inhibitor of human cytochrome P-450 (CYP1B1) [84]. This property of PMS could have diversified its role in various cancers, because Cytochrome P450 (P450) 1B1 was expressed in various human tissues, including prostate, ovary, uterus and mammary gland. Pentamethoxystilbene also inhibited P450 1A1, which was considered to be a vital enzyme associated with tumor initiation. PMS inhibited the activity of human P450 1A1 in a mixed-type manner with a K_i_ of 0.05 μM, and the mechanism was reversible [84]. A quick overview of methoxylated resveratrol derivatives was summarized in Table 1.

## 3. Hydroxylated Resveratrol Derivatives 

**Figure 3:**
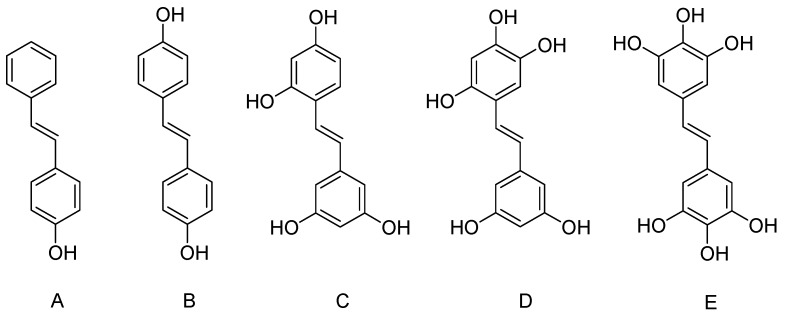
Chemical structure of selected hydroxylated resveratrol derivatives. (**A**) Hydroxystilbene; (**B**) dihydroxystilbene; (**C**) Tetrahydroxystilbene; (**D**) Pentahydroxystilbene; (**E**) Hexahydroxystilbene.

SAR revealed that the addition of 4-hydroxy group in the trans conformation on 4- and 4′-positions of the stilbenic structure (Figure 3) could increase the therapeutic versatility of the parent compound resveratrol.

### 3.1. Dihydroxystilbene 

The pharmacokinetic profiles of *Trans*-4,4′-dihydroxystilbene (4,4′-DHS) were subsequently assessed in rats. Upon I/V administration, 4,4′-DHS had a moderate apparent volume of distribution of the central compartment and a relatively short mean transit time (MTT = 24.1 ± 8.8 min). However, upon an oral administration (10 mg/kg), 4,4′-DHS was absorbed slowly (*t*_max_ 180 or 300 min) with very limited plasma exposure and absolute oral bioavailability (*F* = 2.22 ± 0.72%). On the other hand, when 4,4′-DHS was fully solubilized by hydroxypropyl-β-cyclodextrin, it was absorbed rapidly (*t*(max) 30 or 45 min) with a more than 15-fold increase in maximal plasma concentration (C(max)), plasma exposure (AUC(0→last)) and bioavailability (*F* = 36.3 ± 4.8%). Aqueous solubility was found to be a barrier to oral absorption of 4,4′-DHS while such barrier could be overcome by solubilizing 4,4′-DHS with hydroxypropyl-β-cyclodextrin. The statistical comparison showed that 4,4′-DHS was better than resveratrol from the perspective of pharmacokinetics [86].

#### 3.1.1. Chemotherapeutic Activity 

4,4′-dihydroxy-*trans*-stilbene (2 OH group in 4′- and 4-positions) a resveratrol derivatives, was synthesized to increase its growth inhibitory potential. 2,3- and 4,4′-dihydroxystilbene was reported to inhibit the metastasis and tumor growth in lung cancer. It showed its effect by suppressing the M2 differentiation, which inhibited the STAT3 phosphorylation. The effect of 2,3- and 4,4′-dihydroxystilbene were due to anti-lymphangiogenesis and M2 regulation [87].

*Trans*-4,4′-dihydroxystilbene (4,4′-DHS) was a potent non-toxic chemotherapeutic against neuroblastoma. 4,4′-DHS (Figure 3) in the human neuroblastoma IMR32 cells was shows destabilization of mitochondrial and lysosomal membranes. Its cause lysosomal membrane permeabilization to reduce MMP/caspase-3 activation through the production of cathepsins B, L and D, and the cathepsins [88].

Studies showed that 4,4′-DHS induced a G1-phase arrest, which was associated with high levels of p21 and p53 protein [52]. Furthermore, it could trigger the phosphorylation of Chk (S-phase checkpoint protein) and suppressed tumor-induced neovascularization in colon carcinoma [89]. 4,4′-DHS suppressed human breast cancer cells and inhibited the transformation of fibroblasts more efficiently than the parent compound resveratrol [90]. It could inhibit the two-stage (3-methylcholanthrene plus 12-*O*-tetradecanoylphorbol-13-acetate) cell transformation, inhibited MCF-7 growth, decreased MMP-2 and MMP-9 level and inhibited pRb protein phosphorylation [90,91].

Resveratrol binds to estrogen receptors [92], SAR studies of resveratrol derivatives in estrogen-sensitive breast carcinoma cells showed that minor changes in resveratrol derivatives structure lead to distinct binding to estrogen receptor alpha [91]. 3,4′-Dihydroxystilbene, 4,4′-dihydroxystilbene and 4-hydroxystilbene were reported to bind to estrogen receptor alpha [93]. These different binding activity of resveratrol derivatives to estrogen receptor alpha resulted in various biological activities in estrogen-sensitive cancer cells. Furthermore, 4,4′-DHS administration in HL-60 promyelocytic leukemia cells showed higher antioxidant and cytotoxic activity than resveratrol [94].

#### 3.1.2. Cardioprotective Activity

4,4′-DHS was reported to reduce the production of endothelin-1 (ET-1) and preproendothelin-1 (PpET-1) mRNA levels in human endothelial cells without affecting the transcriptional level [95]. 4,4′-DHS actively inhibited the zinc binding metalloendopeptidase (endothelin converting enzyme-1), which was responsible for synthesis and release of endothelin-1 [96]. 4,4′-DHS demonstrated an active endothelin-converting enzyme-1 (ECE-1) inhibitor than the available ECE inhibitors [95].

4,4′-DHS exhibited great potential in protecting against ROS production and hemin-induced lipid peroxidation. 4,4′-DHS showed a dose-dependent inhibition of the amidinopropane hydrochloride (AAPH)-induced LDL peroxidation in the absence of endogenous antioxidants. Prior to AAPH-induced LDL peroxidation 4,4′-DHS administration led to prolong inhibition time of the native LDL peroxidation. The overall inhibition time was the summation of inhibition induced by 4,4′-DHS and native LDL. It was observed that the addition of 4,4′-DHS inhibited the malondialdehyde (MDA) production and enhanced the inhibition time of native LDL. The antioxidant behavior of resveratrol and its derivatives was calculated by their inhibition time, and adopted the categorization of antioxidant as 3,4-DHS > 4,4-DHS > resveratrol~2,4-DHS > 3,5-DHS respectively [97,98]. The mentioned activities of 4,4′-DHS showed that it had great potential to be developed as a potent drug for the prevention and treatment of cardiovascular and related diseases.

### 3.2. Tetrahydroxystilbene 

*Trans*-3,3′,4,5′-tetrahydroxystilbene (3,3′,4,5′-THS), a natural analogue of resveratrol, had multiple biological functions. 3,3′,4,5′-THS metabolites were piceatannol conjugates, *O*-methyl piceatannol, and its conjugates, whereas, resveratrol metabolites were only conjugates. Studies showed that the AUC for 3,3′,4,5′-THS, resveratrol, and their metabolites increased in a dose-dependent manner. The AUC for total piceatannol was less than that for total resveratrol. The greater AUC for 3,3′,4,5′-THS was a result of its higher metabolic stability [99]. 3,3′,4,5′-THS predominantly eliminated via non-urinary routes and were highly distributed into tissues and were highly extracted by the liver. The detectable plasma half-lives of these appeared to be relatively short. However, utilizing urinary concentration-time data, much longer elimination half-lives were evident. The estimates of oral bioavailability characterize these stilbenes as poorly bioavailable compounds [100].

#### 3.2.1. Cardioprotective Activity 

*Trans*-3,3′,4,5′-tetrahydroxystilbene (3,3′,4,5′-THS) showed promising effects as a therapeutic target for cardiovascular diseases (CVD). Its role was extensively investigated from the past decade by pharmacologist and chemist in the context of CVD [101].

3,3′,4,5′-THS (Figure 3) prevented atherosclerosis by its potent antiinflammatory behavior through suppressing proinflammatory cytokines IL-13, TNF-α and spleen tyrosine kinase in human pulmonary artery endothelial cells [102]. Furthermore, 3,3′,4,5′-THS significantly inhibited Platelet-derived growth factor-βB induced proliferation, and migration of human aortic smooth muscle cells [103]. The smooth muscle cells proliferate and migration played a key role in development and progression of atherosclerosis. 3,3′,4,5′-THS showed stronger therapeutic approach in preventing atherosclerosis than resveratrol.

3,3′,4,5′-THS played a key role in the management of hypertension by increasing the mRNA and protein expressions of endothelial NO synthase (eNOS) mRNA [104]. 3,3′,4,5′-THS is reported to prevent angiogenesis, by blocking adipocyte differentiation via inducing C/EBP homologous protein (CHOP) on the differentiation of preadipocytes [105]. It was characterized by inhibiting fat droplet growth and vascular endothelial growth factor (VEGF) formation. 3,3′,4,5′-THS enhanced the CHOP mRNA expression in HeLa cells and liposarcoma and decreased the production VEGF in Lisa-2 cells and fat accumulation [105,106]. Therefore, tetra was proved to prevent angiogenesis and the risk of obesity.

3,3′,4,5′-THS protected the heart from myocardial ischemia by its antioxidant property. The antioxidant activity of 3,3′,4,5′-THS been studied in rat cardiomyocyte, normal human lung fibroblasts cells, cumene hydroperoxide induced damage cells and fibroblasts cells of the human lung [107]. Lee et al. [108] demonstrated that 3,3′,4,5′-THS increase the expression of the antioxidant enzyme heme oxygenase-1 and its mRNA transcripted in endothelial cells and attenuated cytotoxicity. Furthermore, its inhibits H_2_O_2_ and peroxynitrite induced apoptotic features, cytotoxicity, intracellular ROS and reactive nitrogen species accumulation [109]. Thus 3,3′,4,5′-THS was considered to be an effective therapeutic tools for the management of CVDs.

#### 3.2.2. Chemotherapeutic Activity 

3,3′,4,5′-THS was well reported for its antiproliferative, cytotoxic, hormesis and proapoptotic activity. 3,3′,4,5′-THS was studied in SK-Mel-28 melanoma cells [110], and which showed its effect by inhibiting the cells growth and down regulated the expression of cyclins A, E, and B1. It resulted in suppressing of tumor cell in melanoma in the G2-phase [111]. 3,3′,4,5′-THS was a Janus kinase inhibitor (JAK1) [112], and it was reported to be cytotoxic against prostate cancer cells. JAK1 led to STAT3 activation, which belonged to cytokine signal transduction pathway and found in prostate cancer [113]. It increased the cells number in G1 phase and decreased the activity of cyclin-dependent kinase CDK2 and CDK4 [114]. Similarly, it also increased the cells number in G1 phase, in liver cancer (HCT-116) cell and colon cancer cells (CaCo-2) [115]. It down regulated the expression of Cyclin D1, cyclin B1 and CDK 4 as well as p27Kip1, and enhanced the level of cyclin E [116]. Furthermore, as 3,3′,4,5′-THS was produced by resveratrol during metabolism, so it could also suppress the TCDD-dependent induction of CYP1A1 and CYP1B1 in HepG2 cells [117].

3,3′,4,5′-THS resulted in apoptotic cell death of human leukemia cells (U937) [118]. 3,3′,4,5′-THS treatment with U937 cells resulted in the production of apoptotic bodies, downregulated the expression of Bcl-2 and cIAP-2 (anti-apoptotic proteins) and DNA fragmentation in sub-G1 phase [119]. 3,3′,4,5′-THS showed higher activity against leukemia than the parent compound resveratrol. It also suppressed the cervix cancer by inhibiting COP9 signalosome (CSN)-associated kinases CK2 (IC_50_ = 2.5 μm) and PKD (IC_50_ = 0.5 μm), which could stabilize the tumor suppressor p53 and causes apoptosis [120].

#### 3.2.3. Antimicrobial Activity

Duarte et al. reported the antileishmanial activity of 3,3′,4,5′-THS in various leishmania species and compared its efficacy with Pentostan, the first line clinical antileishmanial drug [121]. 3,3′,4,5′-THS had an LD_50_ value of 5.7 μg/mL, which was vital to gain 50% decrease in extracellular promastigotes viability. Other study reported that 3,3′,4,5′-THS could result in 55% reduction in the viability of major promastigotes and had no toxic effects on the human skin fibroblasts [122].

3,3′,4,5′-THS exhibited antiplasmodial activity on protein tyrosine kinase (PTK) activity in stage-specific plasmodium falciparum grown in both chloroquine-sensitive and -resistant strains [123]. PTK played a key role in the initial asexual maturation of the parasite, tetra inhibited the activity of PTK in trophozoites and schizonts stages. Furthermore, it also inhibited PKT activity during the chloroquine-sensitive and -resistant strains stages [124]. Therefore, 3,3′,4,5′-THS was believed to be an ideal candidate for the development of an antiplasmodial drug. 3,3′,4,5′-THS was also reported to be used as anti-acne because of its antibacterial activity. In strains of propionic bacterium acnes, its showed IC_50_ value of 123 mg/L [125].

#### 3.2.4. Neuroprotective Activity

3,3′,4,5′-THS was reported to increase the viability in primary cortical neurons, decreased the loss of dopaminergic neurodegeneration, and improved climbing scores in transgenic Drosophila-expressing human G2019S. 3,3′,4,5′-THS played a significant role in treating Parkinson′s disease by suppressing the mutation of leucine-rich repeat kinase-2, the common cause of Parkinson′s disease [126]. 3,3′,4,5′-THS was reported in Alzheimer model of Abeta-induced PC12 cells. Administration of 3,3′,4,5′-THS prevented apoptosis and inhibits ROS intracellular accumulation in PC12 cells with beta-amyloid. It also suppressed the caspase-3 activation, internucleosomal DNA fragmentation and nucleus condensation [127].

3,3′,4,5′-THS was a Serotonin inhibitor it reduces Serotonin uptake. Serotonin was a neurotransmitter that played a key role in numerous neuropathological disorders, including depression, mood, hypertension and cellular signaling. 3,3′,4,5′-THS reduced the Syk-activity and Tyr-phosphorylation of anti-SERT-immuno-stained proteins in membrane extracts [128]. 3,3′,4,5′-THS has the potential to target various enzymes and proteins which led to its neuroprotective role and regulated the learning capacity in aging. Studies showed that it suppressed the formation of Aβ fibrils and protected the hippocampal cells against β-amyloid (Aβ)-induced toxicity [129]. Furthermore, it inhibited binding sites of choroid plexus, which secreted transthyretin protein and led to modulation of Aβ aggregation [129].

### 3.3. Hexahydroxystilbene

3,3′,4,4′,5,5′-hexahydroxy-*trans*-stilbene (3,3′,4,4′,5,5′-HDS) is a synthetic resveratrol derivative, advertised as a candidate drug highly effective against various pathophysiological process. The presence of additional hydroxy (–OH) groups in the highly reactive position ortho can enhance its biological activities [130]. Another property exacerbated by the additional ortho-hydroxyl groups was the generation of mitochondrial reactive oxygen species (ROS), especially superoxides, whose deleterious activity might underlie either anti-proliferative or pro-apoptotic capabilities of 3,3′,4,4′,5,5′-HDS [22].

#### 3.3.1. Chemotherapeutic Activity

3,3′,4,4′,5,5′-hexahydroxy-*trans*-stilbene (3,3′,4,4′,5,5′-HDS) were reported to suppress growth and induce apoptosis in numerous malignancies, including colon, breast, leukemia, melanoma and glioma cells. 3,3′,4,4′,5,5′-HDS (Figure 3) potentially suppressed the growth of gastrointestinal cancers cells. The presence of ortho-hydroxyl groups in 3,3′,4,4′,5,5′-HDS structure increased its cytotoxicity and biological activities. 3,3′,4,4′,5,5′-HDS metastasized numerous cancers into peritoneal cavity [130]. In human breast cancer cells, 3,3′,4,4′,5,5′-HDS induced cytotoxicity by apoptosis, of cell proliferation and clonogenic survival [131,132]. Studies showed that 3,3′,4,4′,5,5′-HDS activate p53 accumulation, inhibited mitochondrial super dismutase potential and caspase activation [131,133]. In HT29 human colon cancer cells, 3,3′,4,4′,5,5′-HDS induced apoptosis and caused cell cycle arrest. Furthermore, it showed significant alteration of the deoxyribonucleoside triphosphate (dNTP) pool balance in cancerous cells, which leads to blockage of DNA synthesis in rapidly growing cancer [133].

3,3′,4,4′,5,5′-HDS was studies in T cell leukemia Jurkat cells, its showed cytotoxic activity against the cancer cells having a IC_50_ value of 33.4 μM and increased activity of caspase 3 and 9. The presence of ortho-dihydroxy structures enhanced the antioxidant property of resveratrol derivatives. 3,3′,4,4′,5,5′-HDS was reported to possess stronger antioxidant activity and inhibited the incidence of DNA single-strand breaks upon exposure to H_2_O_2_ in leukemia cell lines [134].

3,3′,4,4′,5,5′-HDS significantly impaired melanoma progression in a mouse model (cell line M24met). 3,3′,4,4′,5,5′-HDS induced G2/M arrest and led to apoptosis by upregulation of p21 and downregulation of CDK-2 [135]. Furthermore, it induced phosphorylated p53 and various proteins, including mismatch homology 6 (MSH6), mismatch homology 2 (MSH2), and MutL homolog 1 (MLH1) [15,135]. 3,3′,4,4′,5,5′-HDS is a potent chemotherapeutic target for various types of cancer, and it was proved to be more potent than the parent compound resveratrol. Studies showed that the combination of the conventional chemotherapeutic regimen with 3,3′,4,4′,5,5′-HDS could further increase its beneficial effects [136].

#### 3.3.2. Anti-HIV

Numerous hydroxy-moieties in the stilbene ring of resveratrol possessed stronger anti-HIV (human immunodeficiency virus) activities than parent compound resveratrol [137,138]. Among various methoxylated and hydroxylated resveratrol derivatives, 3,3′,4,4′,5,5′-HDS was reported to be the most potent and promising therapeutic target for HIV at the initial step in HIV replication before the fusion to host cells and reversed transcription [139]. It showed potent anti-HIV activity against various HIV variants, with EC_50_ values in the low μM range. The 3,3′,4,4′,5,5′-HDS mechanism of action against HIV was demonstrated to be independent of tropism, and was different from other resveratrol derivatives as it blocked the HIV at entry step. Furthermore, 3,3′,4,4′,5,5′-HDS didn’t possess toxicity, so it had the potential to use as adjuvant therapy for HIV treatment and as a microbicide to block HIV transmission [139].

#### 3.3.3. Selective COX-2 Inhibitor 

Hydroxylated resveratrol derivatives was a promising candidate and characterize a new and significantly high class of selective COX-2 inhibitors [128,131]. Docking studies on cyclooxygenase (COX-1 and COX-2) protein structures show that 3,3′,4,4′,5,5′-HDS could bind to the binding sites of prostaglandin endoperoxide synthase. The binding ability of 3,3′,4,4′,5,5′-HDS to the cyclooxygenase enzyme is much more promising and the selectivity index was higher than the celecoxib (clinically uses COX-2 inhibitor). However, other classes of resveratrol derivatives, including methoxylated, halogenated, etc., showed poor or no COX-2 inhibitors with no specificity for COX-2 [131]. Hydroxylated resveratrol derivatives represented a unique class of highly selective COX-2 inhibitors and promising candidates for in vivo studies [131]. Further in vivo studies with different animals models and SAR would be helpful to further highlighted the role this class of derivatives to initiate future clinical trials. A quick overview of hydroxylated resveratrol derivatives was summarized in Table 2.

## 4. Halogenated Derivatives of Resveratrol

**Figure 4:**
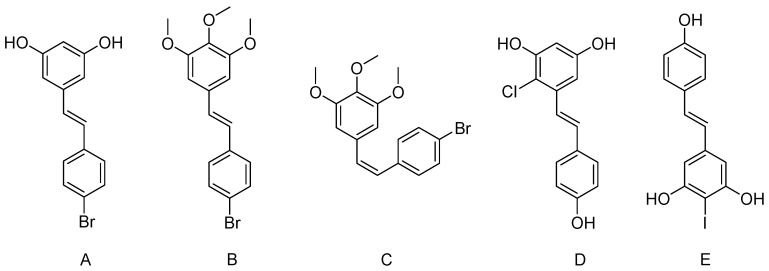
Chemical structure of selected halogenated resveratrol derivatives. (**A**) 4′-Bromoresveratrol; (**B**) 3,4,5-Trimethoxy-4′-bromo-*trans*-stilbene (BTS); (**C**) 3,4,5-Trimethoxy-4′-bromo-*cis*-stilbene (BCS); (**D**) 2-Chlororesveratrol; (**E**) 4-Iodoresveratrol.

The addition of halogen group to the stilbene structure (Figure 4) could increase the therapeutic versilititlity of resveratrol compound and gave diverse and interesting benefits.

### 4.1. Chemotherapeutic Activity 

Several fluorinated resveratrol derivatives had been reported for potency equal to resveratrol. A fluorinated analog (*E*)-3,5-di-fluoro-4′-acetoxystilbene displayed an even greater anticancer activity than the parent compound resveratrol. Similarly, this compound had been reported for synergistic activity of epirubicin when used in combination [140]. Another halogenated derivative of resveratrol “3,4,5-trimethoxy-4 V-bromo-*cis*-stilbene (BCS)” (Figure 4) had been found more effective than its corresponding trans -isomer and resveratrol on the inhibition of lung cancer cell growth by BCS via G2/M phase cell cycle arrest [141]. 4′-bromo-resveratrol could inhibit Sirtuins significantly than resveratrol, and it also inhibited rather than activated sirtuins. It acted on nicotinamide adenine dinucleotide (NAD^+^) and substrate peptide binding, as the bromo-phenyl group was not recognized by the binding site, and resulted in sirtuins inhibition [142]. It was worthy to note that sirtuins is associated with various pathological conditions, including apoptosis, aging, transcription, and inflammation.

### 4.2. Cardioprotective Activity

The deposition of amyloid transthyretin in peripheral nerves and hearts plays a mechanical role in reducing organ function. Halogenated derivatives of resveratrol not only stabilized the native tetramer but they could modify the quaternary structure of monomeric transthyretin in solution by accelerating monomeric transthyretin aggregation or by inducing tetramer formation [143]. In both cases, there was a decrease in the concentration of the monomeric transthyretin in solution, the precursor of the cytotoxic species, resulting in the prevention of cell damage.

### 4.3. Free Radical Scavenging Activities 

Resveratrol derivatives with bromo, iodo and fluoroethyl groups at various positions had been reported for increased inhibition of free radical like diphenylpicrylhydrazyl (DPPH). These halogenated resveratrol derivatives revealed lower IC_50_ values for the mentioned fee radical as compared to parent moiety of resveratrol [24].

### 4.4. Antimicrobial Activity 

The antimicrobial activity of resveratrol and its halogenated derivatives has been reported against Gram-positive bacteria (*S. aureus*), Gram-negative bacteria (*E. coli*) and yeast (*C. albicans*). The study shows that 2-chloro-resveratrol and 2-bromo-resveratrol (Figure 4) were most potent against C. albicans when compared with the resveratrol and fluconazole [24]. The addition of iodide to the stilbene backbone increase its antibacterial activity [144]. A quick overview of halogenated resveratrol derivatives is summarized in Table 3.

## 5. Conclusions

Resveratrol has been proved to possess remarkable health benefits. However, the low bioavailability of resveratrol encumbers its therapeutic application. So, the structural modification of resveratrol has received more particular attention, and researchers developed many resveratrol derivatives. In this review, we investigated the chemical structure of resveratrol derivates and their pharmacological activity and found the methoxylated, hydroxylated and halogenated resveratrol derivatives exhibited favorable therapeutic potential. The further structure–activity relationship study of resveratrol derivatives would be helpful for their practical applications.

## Figures and Tables

**Table 1:** Overview of methoxlated resveratrol derivatives, their therapeutic benefits, mechanisms and effects on various biomarkers.

Methoxylated Resveratrol Derivatives	Mechanism	Therapeutic Benefits	Comparison with RSV	Effect on Biomarker	References
Pterostilbene	Suppressing various signal transduction pathways. Inhibiting ethanol-induced oxidative DNA damage.	Antioxidant. Chemotherapeutic activity in pancreatic, melanoma, leukemia, breast, lung and gastric cancer. apoptosis, Anti-invasive, antimetastatic, anti-inflammatory. Manage hypertension and vessel diameter. Antidiabetic.	Increased lipophilicity over resveratrol.	COX-2 ↓, iNOS ↓, NF-ĸB ↓, AP-1 ↓, MMP-9 ↓, Akt ↓, p38 MAPK ↓, TNF-α ↓, IL-1b ↓, IL-6 ↓, COX-1 ↓, COX-2 ↓, LDL ↓, HDL ↑,	[30,33,36,40,42,44,49,85]
Trimethoxystilbene	Down-regulating phosphatidylinositol 3-kinase (PI3K)/AKT signaling. Blockade of neovascularization by antiangiogenic compounds. Immature vessel disruption by vascular-targeting agents. Bundling of microtubules and speckle-like structures of DCLK1—microtubule complexes.	Chemotherapeutic role against breast, lung and liver cancer. Vascular-targeting agent. Anti-HCV	30 to 100 time more potent than the resveratrol in inhibiting endothelial cell proliferation and morphogenesis.	NF-ĸB ↓, AP-1 ↓, MMP-2 ↓, MMP-9 ↓, VEGFR2 mRNA expression ↓, β-catenin ↓.	[54,55,56,57,61]
Tetramethoxy stilbene	Perinuclear mitochondrial clustering by membrane permeability transition, release of cytochrome c into the cytosol and DNA fragmentation. Anti-angiogenic activity by Inhibiting the phosphorylation of multiple downstream signaling components of VEGFR2.	Inhibiting the growth of various cancers, including colon, prostate, ovarian and liver. Inhibits Cytochromes P450. Manage hypertension and cardiac fibrosis.	High potency and bioavailability than resveratrol.	Bax ↑, Bcl-2 ↓. Akt ↓, FAK ↓, c-Src ↓, mTOR ↓, p70S6K ↓ and Erk1/2 ↓.	[65,68,69,70,73,75,76,77]
Pentamethoxystilbene	Induces G1 cell-cycle arrest and G1 cell-cycle regulatory proteins.	Inhibiting the growth of breast and colon cancer. Inhibits Cytochromes P450.	Potent inhibition of cell growth than resveratrol	cyclin D1 ↓, D3 ↓ and E ↓, CDK2 ↓, 4 ↓ and 6 ↓, ERK1 ↓, p38 MAPK ↓.	[81,83,84]

Increase (↑), Decrease (↓), Cyclooxygenase-2 (COX-2), inducible nitric oxide synthase (iNOS), nuclear factor kappa-light-chain-enhancer of activated B cells (NF-κB), Matrix Metalloproteinase 9 (MMP-9), P38 mitogen-activated protein kinases (p38 MAPK), tumor necrosis factor alpha (TNF-α), Interleukin 1 beta (IL-1b) , Interleukin (IL-6), Low density lipoprotein (LDL), High density lipoprotein (HDL), Matrix Metalloproteinase 2 (MMP-9), vascular endothelial growth factor receptor 2 (VEGFR2), Focal Adhesion Kinase (FAK), Proto-oncogene tyrosine-protein kinase Src (c-Src), mammalian target of the rapamycin (mTOR), p70 ribosomal S6 kinase (p70S6K), Extracellular Signal-Regulated Kinases ½ (Erk1/2), Cyclin-dependent kinase 2 (CDK), Extracellular signal regulated kinase 1 (ERK1) and hepatitis C virus (HCV).

**Table 2:** Overview of hydroxylated resveratrol derivatives, there therapeutic benefits, mechanism and effect on various biomarkers.

Hydroxylated Resveratrol Derivatives	Mechanism	Therapeutic Benefits	Comparison with Resveratrol	Effect on Biomarkers	Reference
Dihydroxystilbenes	Inhibits cancer progression by metastasis and tumor growth via G1-phase arrest. Reduced total endothelin-1 secretion and endothelin-1 messenger RNA (mRNA) levels in human endothelial cells. Active in protecting against hemin-induced lipid peroxidation and ROS production.	Chemotherapeutic against lung and breast cancer. Manage vascular abnormalities. Antioxidant.	More active than resveratrol.	p21↑, p53↑, VEGF↓, LDL↓	[87,90,92,93,97,98]
Tetrahydroxystilbene	Inhibits oxidation of LDL-c in plasma, platelet aggregation, and inflammation. Antiproliferative, cytotoxic, hormesis and proapototic aginist cancer cells. Suppress the mutation of leucine-rich repeat kinase-2, inhibits the Serotonin uptake.	Management of atherosclerosis, hypertension, myocardial ischemia. Chemotherapeutic activity against liver, leukemia and cervix cancer. Antioxidant. Antimicrobial. Effective in Parkinson and Alzheimer.	Stronger antioxidant and tumor suppressing activity than resveratrol.	Bcl-2↓, COX 2↓, LDL↓, AMPK↑, NF-κB↓, Cyclin D1↓, PTK↓.	[101,111,112,121,123,126,127,129]
Hexahydroxystilbene	Cause a dysbalance of intra-cellular deoxyribonucleoside triphosphates. Inhibition of viral attachment and reverse transcription to host cells before replication. Inhibits prostaglandin-endoperoxide synthase.	Inhibit growth in numerous malignancies, including breast and colon cancers ,leukemia, melanoma, and glioma cells. Anti HIV-1. selective COX-2 inhibitor, Antioxidant.	6600-fold higher antiradical activity than resveratrol, most effective free radical scavenger of all resveratrol analogues, higher anti-HIV-1 activity than resveratrol.	NF-κB, p53↑, COX-1↓ and COX-2↓, SOD↑, SA-β-gal↓, SIRT1 expression↑.	[130,131,133,135,137]

Increase (↑), Decrease (↓), vascular endothelial growth factor (VEGF), Low density lipoprotein (LDL), Cyclooxygenase-2 (COX-2), Adenosine monophosphate-activated protein (AMPK), nuclear factor kappa-light-chain-enhancer of activated B cells (NF-κB), protein-tyrosine kinase (PTK), Cyclooxygenase-1 (COX-1), Superoxide Dismutase (SOD), Senescence-associated beta-galactosidase (SA-β-gal) and sirtuin 1 (SIRT1).

**Table 3:** Overview of Halogenated resveratrol derivatives, there therapeutic benefits, mechanism and effect on various biomarkers.

Halogenated Resveratrol Derivatives	Mechanism	Therapeutic Effects	Comparison with Resveratrol	Reference
(*E*)-2,6-dibromo-4-(3,5-dibromostyryl)phenol	stabilize the native tetramer of amyloid transthyretin and modify the quaternary structure of monomeric transthyretin in solution	Cardiprotective effect	Higher bioavailability than resveratrol	[143]
(*E*)-3,5-di-fluoro-4′-acetoxystilbene	Inhibiting upregulation of cellular transporter proteins belonging to the ABC superfamily	antiproliferative	greater anticancer activity than resveratrol	[140]
3,4,5-trimethoxy-4′-brom-*cis*-stilbene	Suppressing the growth of cancer cell through G2/M phase cell cycle arrest	inhibitor of the growth of lung cancer cells	more effective than in suppressing tumor growth than resveratrol	[141]
4′-Bromo-Resveratrol	potently inhibited Sirt1 and Sirt3 by overlapping through extending its bromo-phenyl group at the active site	Therapeutic effects in aging, transcription, apoptosis, inflammation related diseases	inhibited Sirt3 with much higher potency than resveratrol	[142]
2-bromo-resveratrol	unknown	Antimicrobial, Antiproliferative	3 fold lower MIC values against *C. albicans* than resveratrol	[144]
2-chloro-resveratrol	unknown	Antimicrobial, Antiproliferative	30 fold lower MIC values against *C. albicans* than resveratrol	[144]

Sirtuin-3 (Sirt3), Minimum Inhibitory Concentration (MIC).
